# Marginal bone loss in implant placement among Indian patients with fresh extraction socket

**DOI:** 10.6026/97320630019770

**Published:** 2023-06-30

**Authors:** Manu Sharma, Srinivasulu Pabbaraju, Sabari Murugesan, Praveen Gangadharappa, Shyamalima Bhattacharyya, Subhash Chandra Pankaj

**Affiliations:** 1 Department of Oral and Maxillofacial Surgery, GMC, Saharanpur, India; 2 Department of Oral and Maxillofacial Surgery, Adiparasakthi Dental College and Hospital, Kanchipuram, Tamil Nadu, India; 3 Department of Restorative Dental Sciences, College of Dentistry, Jazan University, Jazan, Kingdom of Saudi Arabia; 4 Department of Prosthodontics, College of Dentistry, Jazan University, Jazan, Kingdom of Saudi Arabia; 5 Department of Public Health Dentistry, Government dental college, Dibrugarh, India; 6 Department of Prosthodontics, Government Dental College, Raipur, Chattisgarh, India

**Keywords:** Immediate implant, Fresh extracted socket, Marginal bone loss

## Abstract

It is of interest to examine the Marginal Bone Loss (MBL) around a single titanium implant that was immediately loaded and implanted in a healed or new extraction site in the maxilla over a period of five years. This study involved 36 participants (21 men and 15 women, with an average age of 31 years), all of whom needed to have a single implant placed in the front maxillae. The average marginal bone loss (MBL) in extraction sockets increased by 0.27±0.18 mm after a year, 0.26± 0.17 mm after three years, and 0.21 ±0.18 mm after five years. The mean change in MBL for the group of healed ridges was 0.27± 0.18 mm for one year, 0.22±0.18 mm for three years, and 0.21± 0.17mm for five years. Thus, implants loaded immediately and positioned in either healed ridges or extraction sockets had a similar effect on the local bone.

## Background:

Dental implants are a critical surgery for restoring function for people who are either entirely or partially edentulous. [1] Dental implant treatment has traditionally involved inserting a dental implant in a bone that has healed and then allowing it to fully recover. Immediate placement of dental implants in extraction sockets has become a secure and practical therapeutic alternative as a result of improvements in implant surgery throughout time. [2] A few of the obvious benefits of immediate implant insertion include better alveolar and soft tissue morphology preservation, fewer surgical procedures being required, a quicker recovery period, less stress on the patient, and instant benefits. [3] Many drawbacks of implant treatment have been observed in addition to the benefits, such as the necessity of using regeneration methods. Bone grafts and barrier membranes may become even more necessary due to the incompatibility between the morphology of the dental implant and that of the extraction socket. [2] In cases of periapical lesions, regeneration of bone, insertion of implant and tooth extraction can be achieved in a single surgery by placing the dental implants in extraction socket. [4] Immediate implant administrations, in contrast to the standard approach, do not permit the bodily tissues to complete the infection control. The technique's drawback is that if an infection in the socket continues, the implant may get contaminated during the first healing process.[2] Immediate implant administrations, in contrast to the standard approach, do not permit the bodily tissues to complete the infection control. [4] Immediate implant applications, in contrast to the conventional method, do not permit the body tissues to finish the infection control. During implant surgery, dangerous germs may still be present at the extraction sites despite intensive irrigation.[5] To lessen the inflammatory reaction, all soft tissue remnants in sockets must be properly curettage, including granulation tissues.[3] Prophylactic antibiotic use to minimize infection risk, antiseptic irrigation for decontamination in mechanically difficult-to-reach areas, and the use of lasers for extraction socket debridement before immediate implant placement in extraction sockets are a few techniques that have been discussed in the literature. [6] Despite the fact that the use of systemic antibiotics during dental implant procedures is debatable, it has been made clear that different antibiotic kinds and dosages are recommended in trials with a comparable design. [3, 7] Antibiotics are generally helpful in reducing failure in dental implant surgery, according to a thorough research of the impact of systemic antibiotic administration on difficulties in the installation of dental implants. [8] Before placing an immediate implant in a contaminated location, extraction sockets should be irrigated with chlorhexidine solutions to drastically reduce contamination levels. [9,10] Despite recent studies showing great success rates for immediate implant placement in sockets with chronic periapical illness, the risks of the application are still being discussed in clinical praxis. [11] Marginal bone loss (MBL) is a surgical and prosthetic condition that can be influenced by a number of surgical and prosthetic factors, including freshly extracted socket. [12] Hence, it is of interest to examine the MBL around a single titanium implant that was immediately loaded and implanted in a healed or new extraction site in the maxilla over a period of five years.

## Methodology:

The conduct of this study was done at GMC, Saharanpur, UP, India and was guided by the Helsinki Declaration for the Protection of Human Subjects. Individuals needed to fulfill the following criteria in order to be eligible: age range of 20 to 60; only need one dental implant in the front maxilla; sufficient amount of bone volume to support an implant; and the presence of both mesial and distal natural teeth at the implant site. For the immediate loading protocol, implants with an initial stability of at least 32 N/cm are required if the apical bone height is greater than 5 mm from the extraction socket. This study did not examine any previous bone grafting or bone regrowth close to the implant site. Exclusion criteria included chronic peri-apical lesions of endodontic origin in the implant site, systemic diseases that preclude oral surgery, smoking more than 20 cigarettes per day, uncontrolled periodontal disease, insufficient bone volume, the need for bone regeneration or augmentation prior to implant placement.

Following these guidelines, this study involved 36 participants (21 men and 15 women, with an average age of 31 years), all of whom needed to have a single implant placed in the front maxillae. The same oral surgeon treated a canine, seven premolars, 21 central incisors, 13 lateral incisors, and other teeth. Twenty implants (group I) were used to replace the ten central incisors, the seven lateral incisors, and the three premolars, and 22 implants (group II) were used to replace the eleven central incisors, the six lateral incisors, the one canine, and the four premolars. These implants were placed into extraction sockets that had been created following the removal of the diseased teeth. Patient bone chips collected during the drilling process were used to fill in any gaps between the implants and the socket. [24] It was possible to temporize right away using the Ti Design or Zir Design abutments, which provided a restorative margin that was about 1.5 mm below the mucosal margin. The abutments were tightened at a rate of 10 N/cm with the aid of a torque controller. Temporary cement was used to hold high-polish temporary crowns in place inside the mouth. The temporary crowns were positioned with clear connections in the maximum intercuspal position and no eccentric or lateral contacts after the excess cement was scraped off. Single sutures were used to modify and secure flaps around cemented restorations.

Patients were given oral hygiene instructions that included mouth rinses (0.12% digluconate chlorhexidine), which should be used for two weeks. One central implant and one premolar implant were lost before the final restoration operation as a result of a group II infection problem. They were left out of the investigation. The temporary crown was removed eight weeks after the implant was inserted, and the abutment was tightened using a torque controller (per the guidelines of the makers). Using standard prosthodontic techniques, a complete ceramic crown was created from a polyvinyl siloxane impression. Self-adhesive cement was used to set the final crown. Using the long-cone paralleling approach, standardized periapical radiographs were taken with the centre beam perpendicular to the alveolar crest. To standardize the process, an occlusal record was added to each X-ray container. Prior to implant placement, when the final crown was set, and then at intervals of one, three, and five years for check-ups, radiographs were collected. All radiographs were converted to digital form, put through the necessary time/temperature processing (a 4 minute bath at 20°C), and then were stored in JPEG format. Using a calibrated 10x magnification and a digital image processing application, the vertical distance between the implant neck and the bone levels was assessed. To prevent operator variances, each radiograph was read by the same oral and maxillofacial radiologist. The baseline variation was followed at the time of implant. The marginal bone level was measured twice, to the nearest 0.1 mm mesial and distal to the implants, after the final crown was cemented at 8 weeks, at 1 year, 3 years, and 5 years of functional loading. For each implant, the average of these two measures was determined.

## Statistical analysis:

The change in marginal bone level between the baseline and the follow-up tests at 8 weeks, 1 year, 3 years, and 5 years after loading served as the primary end variable. To detect significant changes in marginal bone levels over time, a linear mixed model analysis was performed. In this inquiry, the inherent correlation between repeated data collected from the same person is taken into consideration. The paired Student t-test was utilised to compare the MBL at the distal (D) and mesial (M) surfaces at each time point. The level of 0.05 designated statistical significance. STATA version 10.0 and SPSS version 18.0 were used to conduct the analyses.

## Results:

Two implants from group II were lost altogether prior to the final crown cementation; these implants were excluded from the study. We examined increase in marginal bone loss in freshly extracted sockets in five years. The average loss (MBL) in a year was 0.27±0.18 mm to 0.26± 0.17 mm and 0.21 ±0.18 mm after three and five years respectively. On the other hand the mean change in bone loss among healed group was 0.27± 0.18 mm, 0.22±0.18 mm and 0.21± 0.17mm for one three and five years respectively. We observed a significant marginal loss in healed group than the newly extracted implants (p<0.032 vs p<0.630). We observed significant marginal bone loss at mesial side after cementing and 12 months (p=0.008 vs p=0.035) whereas the distal side of implant remained stable in third and fifth year of follow up. In linear mixed model we observed significant bone loss in freshly extracted socket (p<0.031) than healed sockets (p<0.51). We observed a significant effect of implant duration on average bone loss and mesial bone loss (p=0.0045 and 0.0016 respectively) however, no significant effect was found on distal site (p=0.213). A significant statistical difference between the mesial and distal values at T1 was found for all samples (P = 0.0003), but not at later time points, according to the findings of the paired t-test (Table 1,Table 2,Table 3).

## Discussion:

The findings of current study revealed no discernible difference in the rates of bone loss between the two groups. A detailed analysis of the data revealed that the majority of MBL was detected during the first year of loading, and that the rate of bone loss thereafter remained essentially constant at 0.01-0.02 mm/year. It's noteworthy because some bone loss partially stopped after 5 years. Lower MBL concentrations than those seen in our study were also detected in prior studies with comparable observation durations. [14,15] The bone loss for healed sites (group I) in our investigation was roughly 0.266-0.176 mm, but it elevated to 0.78 mm in another study. [16] This difference can be explained by the biological width. [17] The average MBL in other studies using the identical implant devices over a three-year monitoring period was 0.40 ±1.43 mm [18] and 0.40 1±.51 mm [27]. In contrast to what was seen in earlier studies [19] the delay group (group I) at 5 years had a gain of 0.02 mm and the immediate group (group II) had a gain of 0.05 mm in marginal bone level modifications. The bone fill that takes place during osseous healing in the gap between implants and post-extraction sockets is related to the obvious differences between healed sites and extraction sockets. [20] This explains the apparent increase in marginal bone levels or their barely perceptible change during the course of the investigation. [19]

According to prior studies, the Astra Tech Implant System causes interproximal loss of marginal bone levels in healed ridges were 0.266± 0.26 mm after 12 months. It agrees with the conclusions of an earlier loading study. [18] According to Donati *et al* . (2013) [21], mean increases in marginal bone levels for 4.0 mm implants ranged from 0.17 to 0.66 mm and from 0.48 to 1.0 mm for 4.5-mm implants. Other trials that compared bone level changes at immediately loaded versus traditionally loaded implants [22] or at immediate loading versus instantaneous provision [23] found no differences in the interproximal marginal bone levels. This study discovered that implants placed in healed ridges (group I) and extraction sockets (group II) both experienced quick provisional loading and achieved identical levels of interproximal bone-to-implant contact. Mesial surfaces of the implants showed higher MBL than distal regions. This finding might be explained by anatomical characteristics like the interdental septum or incisive fissure, or it might be the result of how stress is distributed along the implant's neck. [24] After five years, it is important to regularly check the difference in marginal bone levels between the mesial and distal sites in order to maintain adequate levels of oral hygiene. The apparent lower rate of MBL may be due to the definitive abutment attachment occurring simultaneously with implant placement without any additional modifications. These findings support past studies that investigate that how abutment disconnections and reconnections affect peri-implant bone resorption. [25] Berglundh *et al* . [26] examined the changes to the marginal bone after implant insertion, abutment connection, and functional loading in 2005. They found that minimal to no changes in bone level followed the most significant bone loss, which happened right after implant implantation and abutment connection. Both our findings and other clinical studies are supported by these outcomes [27] The current study found that placing immediately loaded implants in recently extracted sockets (group II) considerably reduces marginal ridge resorption. This study is subject to a number of limitations due to the enormous number of cases and implants. Additionally, it is currently recommended that the depth of implant insertion be no less than 2-3 mm apical to the neighbouring clinical crown edge. This places some constraints on immediately inserting implants in freshly produced extraction sockets. Furthermore, it is advised against placing the implant abutment interface above the facial crest.

## Conclusion:

Data shows that implants loaded immediately and positioned in either healed ridges or extraction sockets had a similar effect on the local bone. In both healed and freshly extracted extraction sites, functional loading approach with prefabricated abutment seems to retain marginal bone surrounding implant. Within the constraints of this experiment and the small sample size, the association between implant location and final abutment attachment appears to decrease MBL and soft tissue collapse. However, there was no correlation between immediate loading and greater MBL in freshly removed teeth with healed sockets.

## Figures and Tables

**Table 1:** Comparison of Marginal bone loss at different time intervals

***Overall average Mean (SD)***	***Distal Mean (SD)***	***Mesial Mean (SD)***
Healed implant
Cementation of crown at 8 weeks	0.25 (0.17)	0.40 (0.25)	0.71 (0.22)
Cementation of crown at 1 year	0.27 (0.18)	0.42 (0.25)	0.18 (0.23)
Cementation of crown at 3 years	0.22 (0.18)	0.30 (0.22)	0.14 (0.20)
Cementation of crown at 5th year	0.21 (0.17)	0.26 (0.22)	0.13 (0.18)
Immediate implant
Cementation of crown at 8 weeks	0.14 (0.16)	0.21 (0.28)	0.07 (0.15)
Cementation of crown at 1 year	0.27 (0.16)	0.26 (0.22)	0.28 (0.24)
Cementation of crown at 3 years	0.26 (1.7)	0.30 (0.25)	0.24 (0.22)
Cementation of crown at 5th year	0.21 (0.18)	0.21 (0.26)	0.20 (0.25)

**Table 2:** Comparison of marginal bone loss by using linear mixed model

***Distal***	***Mesial***	***p-value***
Cementation of crown at 8 weeks	0.91	0.09	0.53
Cementation of crown at 1 year	0.17	0.02	0.007
Cementation of crown at 3 years	0.08	0.16	0.04
Cementation of crown at 5th years	0.09	0.18	0.08

**Table 3:** Difference between mesial and distal marginal bone loss at different time Intervals

***Cementation of crown at 5th year***	***Cementation of crown at 3rd year***	***Cementation of crown at 1st year***	***Cementation of crown at 8 weeks***
	*Cementation of crown at 5th year *		*Cementation of crown at 3rd year*	*Cementation of crown at 1st year*		*Cementation of crown at 8 weeks*	
	Mean (SD)	p-value	Mean (SD)	p-valueMean (SD)	p-value	Mean (SD	p-value
Healed	0.14 (0.20)	0.09	0.16 (0.31)	0.030.17 (0.32)	0.021	0.15 (0.32)	0.08
Immediate implant	0.09 (0.41)	0.95	0.05 (0.32)	0.530.02 (0.33)	0.83	0.15 (0.31)	0.01
